# SmallBARNA 2026: a kingdom-wide bacterial sRNA resource

**DOI:** 10.1093/nar/gkaf999

**Published:** 2025-10-21

**Authors:** Shusruto Rishik, Leidy Alejandra G Molano, Syed Mohammed Khalid, Tiniko Babalashvili, Fadlilah Nur Hasanah, Md Mobashir Rahman, Pascal Hirsch, Friederike Grandke, Emma S Hoffmann, Tobias W Wolff, Georges P Schmartz, Andreas Keller

**Affiliations:** Chair for Clinical Bioinformatics, Center for Bioinformatics, Saarland University, Saarbrücken 66123, Germany; Chair for Clinical Bioinformatics, Center for Bioinformatics, Saarland University, Saarbrücken 66123, Germany; Chair for Clinical Bioinformatics, Center for Bioinformatics, Saarland University, Saarbrücken 66123, Germany; Chair for Clinical Bioinformatics, Center for Bioinformatics, Saarland University, Saarbrücken 66123, Germany; Chair for Clinical Bioinformatics, Center for Bioinformatics, Saarland University, Saarbrücken 66123, Germany; Chair for Clinical Bioinformatics, Center for Bioinformatics, Saarland University, Saarbrücken 66123, Germany; Chair for Clinical Bioinformatics, Center for Bioinformatics, Saarland University, Saarbrücken 66123, Germany; Chair for Clinical Bioinformatics, Center for Bioinformatics, Saarland University, Saarbrücken 66123, Germany; Helmholtz Institute for Pharmaceutical Research Saarland (HIPS), Saarland University Campus, Saarbrücken 66123, Germany; Chair for Clinical Bioinformatics, Center for Bioinformatics, Saarland University, Saarbrücken 66123, Germany; Chair for Clinical Bioinformatics, Center for Bioinformatics, Saarland University, Saarbrücken 66123, Germany; Chair for Clinical Bioinformatics, Center for Bioinformatics, Saarland University, Saarbrücken 66123, Germany; Chair for Clinical Bioinformatics, Center for Bioinformatics, Saarland University, Saarbrücken 66123, Germany; Helmholtz Institute for Pharmaceutical Research Saarland (HIPS), Saarland University Campus, Saarbrücken 66123, Germany; PharmaScienceHub (PSH), Saarland University Campus, Saarbrücken 66123, Germany

## Abstract

Bacterial small RNA are important context-sensitive regulators of gene expression, especially in highly pathogenic bacteria, often controlling virulence. The number of predicted small RNA (sRNA) entries in public repositories has grown exponentially, contrasting with the linear growth of functionally validated sRNAs. While there are databases maintaining sRNA records from single bacterial species or taxonomic groups, a central repository of bona fide sRNAs for all bacteria with evidence, alignment, and RNA expression information is missing. Such a repository is a critical starting point for both wet lab biologists validating sRNA function as well as bioinformaticians creating new models for sRNA prediction. In this paper, we hand-curate 1117 articles from the literature to find 746 sRNAs that have been confirmed by northern blotting, quantitative polymerase chain reaction (qPCR), mutagenesis, or other functional validation methods. We map these sRNA sequences to QC-filtered bacterial genomic assemblies from NCBI, obtaining 3.8 million hits from 44 789 chromosomes and 10 884 plasmids. Finally, we also quantify these sRNAs in a filtered subset of 5292 isolates with available RNA-seq data from the Sequence Read Archive. The bona fide set, alignment, and expression information is available for download and interactive exploration at https://web.ccb.uni-saarland.de/smallbarna/.

## Graphical Abstract

**Graphical Abstract:**
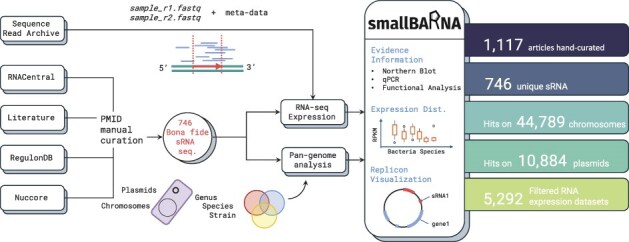

## Introduction

Bacterial small RNAs (sRNAs) are non-coding regulatory molecules 50–500 nucleotides in size that modulate the adaptation of bacteria to stress-related conditions, such as in virulence [1], heat shock condition [2], and immune responses [3]. Some highly pathogenic bacteria, such as *Mycobacterium tuberculosis* [4],*Streptococcus pyogenes* [5], and *Vibrio cholera* [6], use sRNAs to coordinate infection-specific transcriptional changes. There is also emerging evidence of bacteria packaging sRNAs in extracellular vesicles and using them for signal transmission to other bacteria or to the host organism [7–9]. Other than modulating disease, this may also serve as a mechanism of influencing the host gut microbiome. Their context-dependent regulation of the bacterial transcriptome makes sRNAs and their RNA-binding proteins derived from genes such as *Hfq* [10], *ProQ* [11], and *CsrA* [12] promising candidates for manipulating bacterial behavior. For example, targeting an sRNA controlling bacterial biofilm formation [13] or motility [14] may render a pathogenic bacteria vulnerable to the host immune system.

Despite this knowledge, our understanding of sRNAs is still in its infancy. The field of sRNA research is still evolving, with new sRNAs being predicted regularly. The prediction of sRNAs make use of machine learning models trained on features such as k-mer distribution, length, GC content, structural motifs, etc. [15, 16]. Since the dramatic reduction of per-nucleotide cost of next-generation sequencing [17], there has been an exponentially increasing quantity of data available to train models on and predict sRNAs. These predictions have been deposited in public repositories such as RNAcentral [18] and RFam [19], which show the number of sRNA entries in the tens to hundreds of thousands (RNAcentral: 26 162, RFam: 141 050 entries). However, just because an sRNA has been predicted to have a function does not yet guarantee it. The predictions still need to undergo low-throughput validation methods such as mutagenesis followed by functional assays to pin down if there is a function, and if so, what that function is. Unfortunately, the linear rate of low-throughput validation cannot keep up with the exponential growth of sRNA predictions. This would pose no problem if the validation status of the sRNA was included in the publicly available repositories, but this is not the case. As such, it is non-trivial to differentiate functionally validated sRNAs and ones that are merely predicted in these repositories.

Previously, databases such as BSRD [20], sRNABase [21], and sRNADb [22] served as central repositories curating such information about bacterial sRNAs, but they have not been maintained [23]. Databases such as RegulonDB [24] and sRD [25] continue to function but are limited to specific clades, such as *Escherichia coli* and *Staphylococci*. However, sRNAs are found in a wide range of bacterial taxonomic groups that these databases do not include. This leaves a significant gap for a resource curating sRNAs from a broader range of taxa and their corresponding functional information.

SmallBARNA aims to fill this gap by hand-curating the sRNAs from literature that have support from experimental validation into a single database. By also providing alignment and expression quantification information, we hope this will serve as a starting point of analysis for bacterial sRNA studies. Since new sRNAs are constantly being validated, smallBARNA will release mini-updates that keep the contents of the database synchronized with the literature.

## Materials and methods

### Bona fide sRNAs

To create a set of experimentally validated sRNAs (bona fide sRNAs), we manually inspected a list of available articles related to sRNA. To obtain a list of articles to manually curate, we retrieve all linked articles to sRNA entities in both NCBI Nucleotide database (Entrez Direct v.16.2, [26]) and RNAcentral [18] on 27/03/2025. For Nuccore, the following query was used: “(biomol_ncRNA[PROP] OR biomol_transcribed_RNA[PROP]) AND (“small RNA”[All Fields] OR “sRNA”[All Fields] OR “small non-coding RNA”[All Fields] OR “small ncRNA”[All Fields] OR ncRNA[Feature Key]) AND (bacteria[FILT] OR archaea[FILT]) NOT rRNA[Feature Key] NOT tRNA[Feature Key] NOT miRNA NOT snoRNA NOT misc_RNA[Feature Key].” For RNAcentral: (rna_type:ncRNA) AND (tax_string:“bacteria” OR tax_string:“archaea”) AND (description:“sRNA” OR description:“small RNA” OR description:“small non-coding RNA” OR description:“small ncRNA”) NOT rna_type:rRNA NOT rna_type:tRNA NOT rna_type:miRNA NOT rna_type:snoRNA NOT rna_type:snRNA NOT description:miscRNA. Additionally, we used sRNAs from RegulonDB (accessed on the 01/04/2025 through the database’s docker container) [24].

All articles related to the obtained sRNAs entities were manually inspected to retrieve the start, end position, and direction. If the genome accession was available from the paper, the corresponding sequence was extracted. If the genome was not available, the reference genome for the corresponding strain of bacteria was extracted. For certain older manuscripts, the sequence was directly available in the article, and where available, this sequence was extracted. The experimental validation status was extracted by manually reading the papers. Only sRNAs that were experimentally validated by methods such as northern blotting, qPCR, and mutagenesis were included.

Since various groups and manuscripts named their sRNA in a non-uniform way, we kept the original naming in the manuscript to allow users to search through them, but for the database, gave a uniform naming system by taking the first letter of the genus name and the first four characters of the species name, followed by an integer number. For example, an sRNA from *E. coli* would be named sRNA-Ecoli-1.

### sRNA genome mapping

Bacterial and archaeal genomes available from NCBI Datasets [27] were downloaded on 26/05/2025. Genomes were retained only if they had a CheckM [28] completeness of ≥90% and contamination below 5%. For each genome, all available chromosomal and plasmid sequences were downloaded to map against our bona fide set of sRNAs. The local alignment was performed with Bowtie2 [29] using the Snakemake [30] wrapper of Bowtie v6.0.1 using the options “–local –threads 40 -D 20 -R 3 -N 1 -L 20 -i S,1,0.50” to balance sensitivity and specificity. Each taxonomic name associated with a genome assembly was mapped to the lowest available taxonomic identifier (TaxId) from the NCBI Taxonomy [27], while retaining the original custom strain name for completeness.

In addition, BioSample records [27] linked to genome entries were processed for ecosystem identification, including host-associated and environmental categories, using an approach similar to that described previously [31]. Briefly, ecosystems were automatically inferred from the following BioSample attributes: *host*,*host_taxid*,*host_common_name*,*host_animal_breed*,*animal_env*,*local_class*, *soil_type*,*metagenome_source*,*samp_mat_type*,*source_type*,*host_tissue_sampled*,*tissue*,*host_body_habitat*,*isolation_source*,*env_medium*,*env_broad_scale*,*and env_local_scale*. Terms were mapped to a standardized ecosystem ontology. Host-associated ecosystems were further annotated using the NCBI Taxonomy [27] and ETE4 [32]. If a sample was associated with multiple habitats, all relevant habitats were retained. In addition, specific tags were assigned when extra information was identified within the ecosystem-related BioSample attributes: host-disease (“disease”), ecosystem location (“location”), and host traits (“encoded_trait”). These tags were subsequently used in disease and location classification systems (see sections below) when no further information was available. Following automatic classification, ecosystems were manually curated to ensure accuracy and refine assignments.

Host-associated diseases were also automatically extracted from the following BioSample attributes: *host_disease*,*host_disease_outcome*,*host_disease_stage*,*host_health_state*,*phenotype*,*health_state*,*and disease*. Each record was then classified using the Disease Ontology and Symptom Ontology [33, 34]. In cases where multiple diseases were associated with a single sample, all relevant disease terms were retained. As with ecosystems, disease annotations underwent manual curation to validate and enhance the classification process.

Retrieved plasmid sequences were crosslinked with PLSDB [31].

### RNA seq expression

Raw RNA-seq samples were retrieved from the NCBI Sequence Read Archive (SRA) [35] using the following query on 30/06/2025: “(RNA-seq OR RNAseq) AND Transcriptomic AND (“Bacteria”[Organism] OR “Archaea”[Organism]) AND isolate[All Fields] AND “biomol rna”[Properties] AND “library layout paired”[Properties] AND “filetype fastq”[Properties] AND “platform illumina”[Properties].” Only RNA-seq isolates corresponding to organisms with taxIDs linked to previously annotated sRNA genomes were retained. Raw reads underwent quality control using fastp (v0.24.3) [36] with default parameters.

To quantify sRNA expression, each RNA-seq isolate was assigned to one genome from the sRNA genome mapping dataset (see section sRNA genome mapping), selecting the closest available taxonomic match—preferably at the strain level, or otherwise at the species level. When multiple genomes were available for the same organism, they were ranked based on assembly completeness, contamination, and the completeness of associated BioSample metadata, with the top candidate selected.

Quality-controlled reads were aligned to the corresponding genome using Bowtie2 (v2.5.4) [29] with default settings. Read counts mapped to sRNA gene regions were extracted using utility functions from pysam (https://github.com/pysam-developers/pysam) [37]. For downstream analyses, raw counts were normalized to Reads Per Kilobase Million (RPKM).

Taxonomical and BioSample metadata related to each SRA experiment were processed as described in previous sections.

### Comparison of sRNA against known database

To assess the utility of our compiled list of bona fide sRNAs, sRNA sequences from available databases were compared. Specifically, Nuccore and RNA-central sRNA sequences were retrieved as described in the bona fide sRNAs section. Then, sequences were combined, deduplicated using seqkit [38] (v2.8.1, cla: “rmdup –by-seq”), and used to build a local BLAST database [39]. Then, bona fide sRNAs were blasted against the local database (cla: “evalue 0.001 perc_identity 90.0”).

### Software

The data were analyzed using R 4.4.3, Python v3.12.2, ggplot2 3.5.2, pandas v2.2.2, anndata v0.10.8, and tqdm v4.66.2. The database is hosted on a web server set up using the Django Python framework v5.2.1 with PostgreSQL database v17.5. Deployment and stable OS environment are managed by Docker v23.0.6 and Docker Compose v2.29.2. For website development, we employed Bootstrap v5.3.0, JavaScript ES6, DataTables v1.13.7, and jQuery v3.7.1.

## Results

### Validated bona fide set

**Figure 1.**
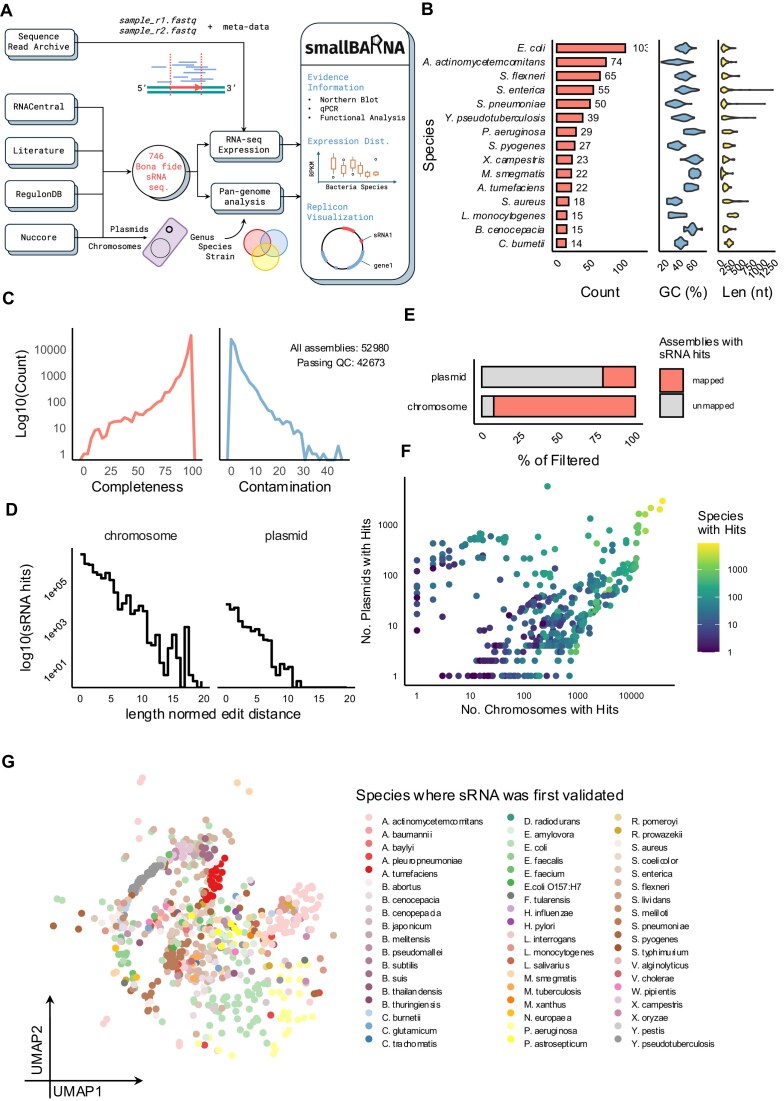
Data distribution for smallBARNA. (**A**) Overview of smallBARNA analysis pipeline. (**B**) Number of validated (bona fide) sRNAs, GC percentage distribution, and length distribution grouped by bacterial species contributing the greatest number of sRNAs from the literature. Only the top 15 species are shown. (**C**) Distribution of completeness and contamination for bacterial genome assembly data downloaded from NCBI and QC filtered. Counts are log10 transformed. (**D**) Log10 transformed length-normalized edit distance distribution of sRNA sequences on bacterial chromosomes and plasmids found by aligning bona fide sRNA sequences using Bowtie2. (**E**) Percentage of chromosomes and plasmids from QC-filtered assemblies that had at least 1 sRNA map onto them (mapped) or did not have any sRNAs map onto them (unmapped). (**F**) Scatterplot of the log-transformed number of chromosomes and the number of plasmids where each bona fide sRNA appeared based on alignment with NCBI genome assemblies. The color corresponds to the log-transformed number of species where each validated sRNA was found. (**G**) UMAP derived from the k-mer sketch (*k* = 31) of each validated sRNA colored by the species where the sRNA was originally validated. Distance matrix for sRNA–sRNA pairs were calculated using the Jaccard distance of the k-mer sketch.

We scraped a total of 1117 publications connected to sRNA from RNAcentral and RegulonDB to obtain 746 sRNA sequences derived from 73 unique bacterial genomes. The number of sRNAs originating from each species follows a power law distribution, as seen in Fig. 1B. *Escherichia coli* contributed the greatest number of sRNA at 103 sRNAs, followed by *Aggregatibacter actinomycetemcomitans* and *Shigella flexneri* at 74 and 65, respectively. The GC content distributions of the sRNA vary based on the species of the sRNA and track with species GC content (*E. coli* ∼50% GC, *Burkholderia cenocepacia* ∼60% GC). The length distribution is less variable with respect to species, with the majority of sRNA falling between 50 and 500 base pairs with a few exceptions. Downstream wet-lab validations of sRNA were available at the northern blot level for 685, RT-PCR for 539, and at qPCR level for 325 sRNAs. Functional validation was available for 532 sRNAs and other miscellaneous validations were available for 125 sRNAs. Of special note, mutagenesis studies were found for 102 sRNAs. Overall, we managed to curate the differential expression evidence of 628 sRNAs from the literature. The sRNAs were primarily validated in medium culture while only 72 sRNAs have been validated in eukaryotic model system such as bronchial epithelial cell line or monocyte cell line.

### Distribution across chromosomes and plasmids

To survey the distribution of our bona fide sRNA set across different bacterial and archaeal genomes, we downloaded the set of 52 980 genome assemblies available in the NCBI Datasets and filtered them by completeness and contamination to obtain 42 673 assemblies (Fig. 1C). These assemblies provide us with 48 598 chromosomes and 51 573 plasmids. Out of these, we found hits using Bowtie2 on 44 789 chromosomes and 10 884 plasmids, totaling 3.8 million sRNA alignments. The distribution of the edit distance between the bona fide sRNA and aligned sRNA sequences shows a wider range of edit distances for chromosomal sRNAs than sRNAs from plasmids (Fig. 1D). Interestingly, while there is a clear bias towards certain species such as *E. coli* in the bona fide dataset, simply because model organisms and highly pathogenic bacteria are more likely to get studied, the alignment results only partially replicate this bias (Supplementary Fig. S1A). While the genera *Escherichia*, *Shigella*, and *Salmonella* align with this bias, many of the top genera where sRNA was found such as *Klebsiella*, *Citrobacter*, and *Enterobacter* do not. Likely, this is because of the large number of chromosomes contributed by these genera from NCBI. While this is partially the case for *Klebsiella*, it is not universally so (Supplementary Fig. S1B). The widespread distribution of sRNA across bacterial chromosomes and plasmids is contrasted by a BLAST search against eukaryotic genomes from NCBI yielding only 4768 hits across 1876 species (Supplementary Table S1).

It is clear that sRNAs are primarily found on chromosomes rather than plasmids (Fig. 1E). Even with approximately the same number of sequences from chromosome and plasmids, at least one sRNA was found on only ∼25% of plasmids, while over 90% of all bacteria chromosomes carry sRNAs. This is despite no obvious bias towards the number of plasmids and chromosomes observed in different genera or species (Supplementary Fig. S1C). However, certain sRNAs likely prefer plasmids rather than chromosomes (Fig. 1F). We see that in general, the higher the number of chromosomes or plasmids an sRNA is found in, the greater the number of species they are shared between. Some are species specific, but the majority of sRNAs are found in a large number of species. Interestingly, the sRNAs form two groups, with one primarily found in chromosomes while the other was primarily found in plasmids. While there are several sRNAs that are only found on plasmids or on chromosomes, the gaps in the scatterplot at either extremes of the axes tells us that once an sRNA becomes sufficiently prevalent (more than ∼1000 chromosomes or ∼100 plasmids), they start to be found in both types of molecules.

The diversity of sRNAs in different bacterial species warrants an exploration of their sequences. Since k-mer signatures have so far proven effective at classifying bacteria [40, 41] as well as sRNA [42], we produced a UMAP of k-mers (*k* = 31) using only the bona fide set in Fig. 1G. While the UMAP does not show a dramatic separation, the sRNAs from different species tend to separate, such as for *Enterococcus faecalis*, *Pseudomonas aeruginosa*, *Yersinia pseudotuberculosis*, *Agrobacterium tumefaciens*, etc. In order to explore if the effect of the taxonomic group of origin or the sRNA sequence effect was greater, we created another UMAP using 5000 randomly sampled sRNAs from the top 20 most highly represented genera. We notice that while large clusters were formed containing sRNAs derived from the same genus such as *Escherichia*, *Streptococcus*, and *Streptomyces*, there were also smaller individual clusters, e.g. from *Escherichia* and *Klebsiella* that remain separated (Supplementary Fig. S1C). Colored by the identity of the sRNA, we notice that these sub-clusterings (and even some of the larger clustering) are originating from individual sRNA (Supplementary Fig. S1D).

### Expression across publicly available RNA-seq datasets

As there are currently thousands of RNA-sequencing datasets available for bacteria, we wanted to explore if our bona fide sRNAs could also be detected in these sets. While RNA-seq can have false positives and no guarantee of function, the presence of an sRNA across multiple samples is evidence that they are at least transcribed in the bacteria of interest. If some functionally validated sRNA are also transcribed in other bacteria, it is a hint that they are serving similar functions there as well.

**Figure 2.**
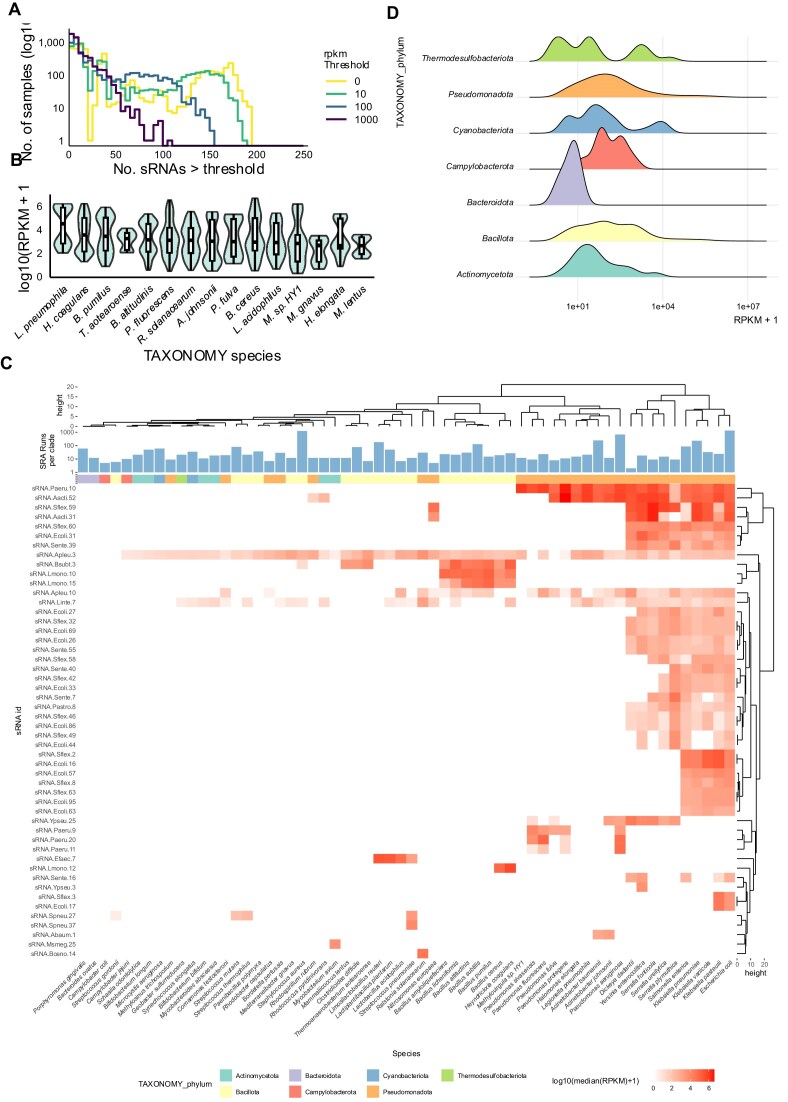
Overview of the RNAseq expression dataset. (**A**) Distribution of number of RNA-seq datasets having a corresponding number of sRNAs with an expression RPKM above a threshold of 0, 10, 100, and 1000 RPKM. Sample counts are log10 transformed. (**B**) Distribution of log10 transformed RPKM mapped to RNAseq datasets obtained from SRA. The expression values are grouped by the species from which the RNAseq data is derived from. The box plots represent the median, first and third quartile of expression. The expression values have been filtered to keep only RPKM > 0. (**C**) Heatmap of log10 RPKM of median sRNA from RNAseq samples from SRA. The rows correspond to the sRNA that was detected and the columns correspond to the genus from where the sRNA median was calculated. (**D**) Distribution of the log10 transformed RPKM expression grouped by bacterial phylum.

Therefore, we filtered 21 445 publicly available RNA-seq datasets based on the taxonomy associate with each isolate, retaining clades whose genomes contained positives sRNA hits, resulting in 5292 samples. A total of 367 sRNAs were scanned across genomes, of which 358 had at least one hit. Among the 5292 evaluated samples, 5137 expressed at least one sRNAs (Fig. 2A). The dataset represents 41 different genera and 60 different species of bacteria, with *E. coli* once again dominating, accounting for 1357 SRA runs (Supplementary Fig. S2A).

The expression of the majority of sRNAs are not ubiquitous, with each species only expressing a small subset of sRNAs (Fig. 2C). While the species-specific distribution of sRNAs spans a wide range (Fig. 2B), sRNA distribution at the phylum level follow clade-specific patterns (Fig. 2D), which remain distinguishable at the family level (Supplementary Fig. S2B). Interestingly, *E. coli* was not in the top 15 species with the highest median sRNA expression, despite being the most studied and sequenced. Similarly, there was only one *E. coli* derived sRNAs in the top 15 sRNAs with the highest median expression (Supplementary Fig. S2C).

A heatmap highlights the sparse nature of sRNA expression (Fig. 2C). We observe that ∼2 out of 3 clusters of sRNA are expressed in members of the *Enterobacterales* family, including species of the genera *Escherichia*, *Klebsiella*, *Enterobacter*, and *Salmonella*. While most of them have been validated in *E. coli*, the other three bacteria genera represent clinically relevant clades where it would be worthwhile to explore the functionalities of sRNA. A small number of sRNA are expressed in almost all genera; notably sRNA-Apleu-3, sRNA-Linte-7, and sRNA-Apleu-10. Closer inspection shows that they are overlapping with tRNAs in the bacteria. The function of these sRNAs have not been extensively validated, as such they represent a few possibilities. They might be tRNAs mistaken as sRNAs. Alternatively, they could be tRNA-derived fragments, which have been found in eukaryotes to be regulating mRNA and are also emerging in the microbe regulatory landscape [43].

## Discussion

In the age of automation, hand-curation might seem unnecessary. But even with Large Language Models (LLMs) swiftly improving, this remains the only way to guarantee accuracy of information. Currently this set of 746 sequences represents the largest hand-curated set of sRNA across the greatest number of prokaryotic species online. This is expected to grow as we incorporate more articles into the database. We endeavored to leverage this specific knowledge into sensitive information as well by creating the largest corpus of aligned sRNAs and expression-quantification as well. The diversity across chromosomes and plasmids from the entire set of NCBI bacterial genomic assemblies are a peek into the tendency for sRNAs to spread across bacteria, likely through horizontal gene transfer enabled by plasmids. Meanwhile, the exploration of k-mer distribution hints at the potential to classify sRNAs into distinct families, similar to other widely studied non-coding RNA such as microRNAs (miRNAs). Similarly, the expression distribution highlight the taxa-specific sparse distribution of sRNA expression across publicly available data, while also highlighting the dominance of expression evidence in clinically relevant members of *Pseudomonodata*,

We hope the scope of our database to be useful for both wet-lab biologists and dry-lab bioinformaticians. Our web interface makes the large-scale alignment and quantification information explorable without the need for programming experience. Biologists looking for sRNA relevant to their bacteria of interest can easily search through our data-tables to see if the supporting information warrants experimental validation. We also include a search function enabled by Bowtie using short sequences to check if they are in already in the database. For more computer-savvy researchers, we make all our data files available. Approximately 3.8 million data points and a clearly defined ground truth set should serve as a convenient starting point for training machine learning models or motif-finding algorithms. When analyzing newly sequenced bacterial RNA datasets, it can also serve as a reference set to quantify the aligned reads.

The drawback of our database also highlights some of the drawbacks of the field in general, e.g. the biased focus on specific classes of bacteria in the field, such as *E. coli*. The most comprehensive functional validations, via mutagenizing specific regions of sRNA or RNA-binding proteins such as *Hfq*and then exposing them to different environmental conditions/stressors to see if there is an impact, have primarily been performed in *E. coli*. Likewise, the interaction with eukaryotic cells has only been demonstrated in a small handful of sRNAs. Currently, no real attempt to classify the sRNAs into families based on their sequences or modification properties has been made, as we feel this is premature at this stage of the database's development as well as our state of knowledge regarding sRNAs.

In conclusion, we hope our database can separate the signal from the noise for bacterial sRNAs and will become the *de facto* updated central repository for this class of non-coding RNA.

## Supplementary Material

gkaf999_Supplemental_Files

## Data Availability

All data are available on the smallBARNA website https://web.ccb.uni-saarland.de/smallbarna/download.
