# Supplementary material for: SmallBARNA 2026: a kingdom-wide bacterial sRNA resource
Source: Nucleic Acids Res. 2025 Oct 21;54(D1):D342–9. doi: 10.1093/nar/gkaf999 (PMC12807594; doi:10.1093/nar/gkaf999)
Supplement: gkaf999_Supplemental_Files [file gkaf999_supplemental_files.zip › Supplementary Figures.pdf]

Supplementary 1

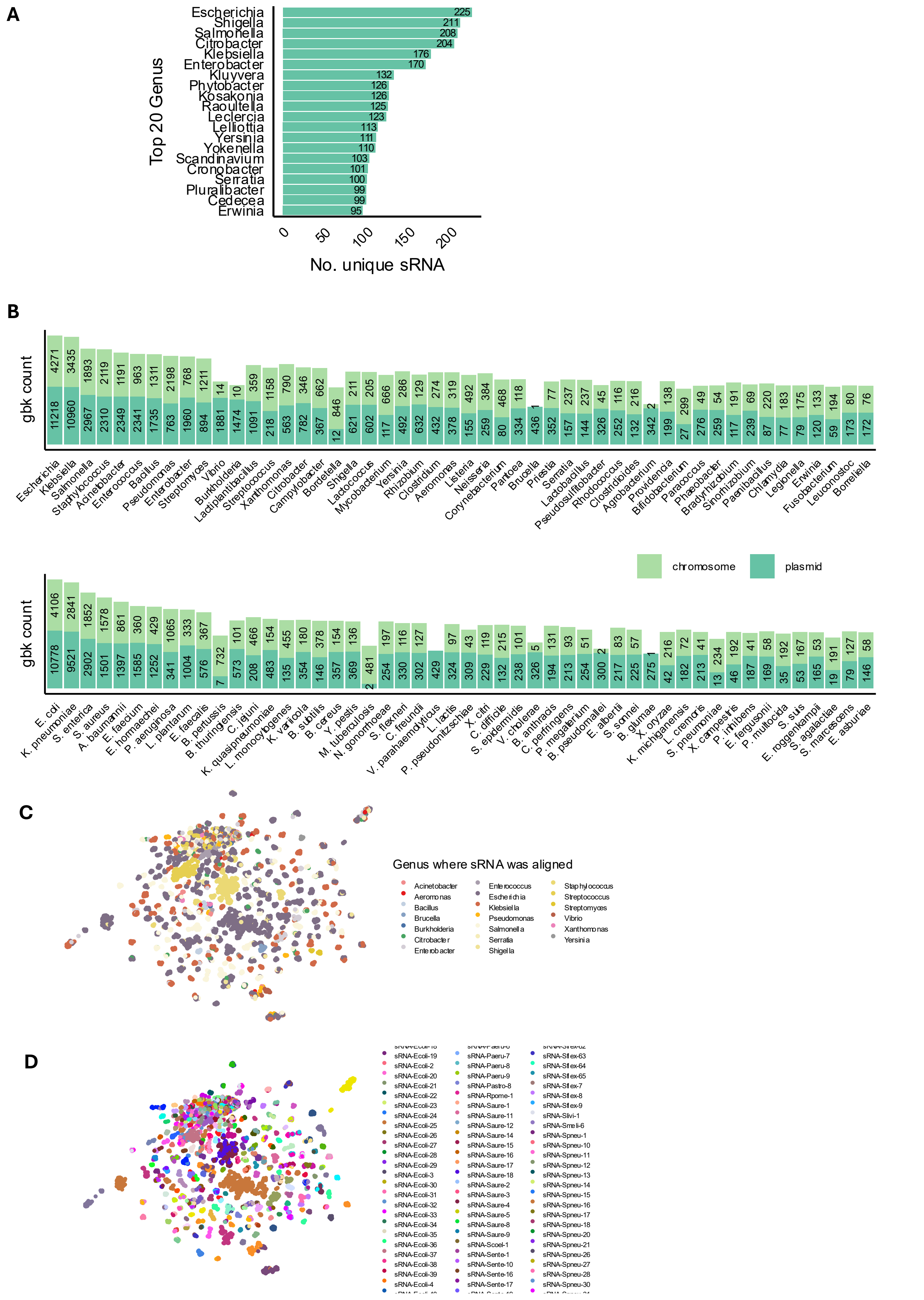

Supplementary 2

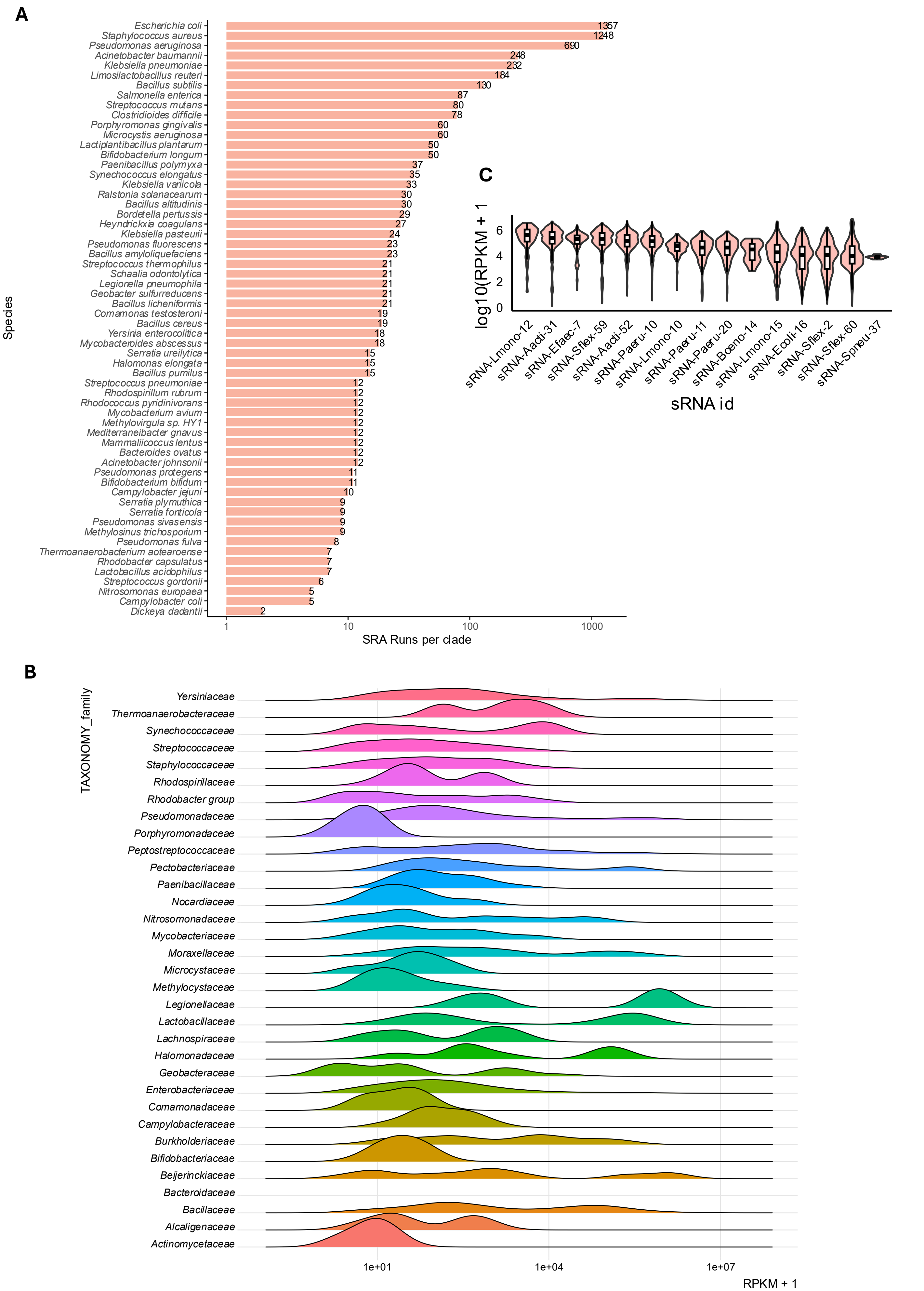

Supplementary Figure 1: a) Top 20 genera ordered by number of unique sRNAs mapping to replicons. **b)** Log scaled number of Genbank files corresponding to chromosome and plasmid for bacterial genera (top) and bacterial species (bottom), top 50 shown. **c)** UMAP of 5000 mapped sRNA sequences sampled from the top 20 most highly represented genera, calculated using Sourmash kmer sketches (k=31, n=500), colored by top n genera of bacteria. **d)** UMAP from c) colored by the bona fide sRNA.

Supplementary Figure 2: a) Log scaled number of RNA-seq runs from Sequence Read Archive per bacterial species **b)** Log scaled expression distribution of sRNA expression split by bacterial family. **c)** Log scaled expression distribution of the top 15 sRNAs.

Supplementary Table 1: Results of comparison of bona fide sRNA sequences against Eukaryotic genomes from NCBI using BLASTN.
